# Phytochemicals Modulating Receptor Tyrosine Kinase Signaling Networks in Endometriosis

**DOI:** 10.3390/ph19091413

**Published:** 2026-09-07

**Authors:** Che-Fang Hsu, Ching-Feng Weng

**Affiliations:** 1 Cellular Oncology Laboratory, Center for Prevention and Therapy of Gynecological Cancers, Department of Medical Research, Hualien Tzu Chi Hospital, Buddhist Tzu Chi Medical Foundation, Hualien 970, Taiwan; 2 Department of Physiology, School of Basic Medical Sciences, Key Laboratory of Functional and Clinical Translational Medicine, Fujian Province University, Xiamen Medical College, Xiamen 361023, China; 3 Leadtek Biomedical, Inc., New Taipei City 235603, Taiwan

**Keywords:** endometriosis, receptor tyrosine kinase signaling, phytochemicals, network convergence, angiogenesis, fibrosis

## Abstract

Endometriosis is a chronic, estrogen-dependent inflammatory condition that is marked by the growth of lesions outside the uterus, along with angiogenesis, fibrosis, immune dysregulation, and pain sensitization. There is now growing evidence that receptor tyrosine kinase (RTK) signaling networks are central to these processes, integrating proliferative, angiogenic, inflammatory, endocrine, and microenvironmental signals. This review summarizes the roles of six major RTK axes in endometriosis and evaluates their receptor-specific mechanisms, therapeutic relevance, and modulation by representative phytochemicals. An English-language literature search of PubMed was conducted with no publication date restrictions using combinations and variations of the following keywords: phytochemicals, endometriosis, EGFR, VEGFR, IGF1R, PDGFR, FGFR, MET, curcumin, resveratrol, epigallocatechin gallate (EGCG), quercetin, naringenin, berberine, apigenin, luteolin, genistein, and baicalein. Additional search terms related to receptor signaling mechanisms, downstream pathways, cellular phenotypes, and therapeutic relevance were also incorporated to ensure comprehensive coverage of the literature. The RTK signaling pathways control stromal activation, lesion survival, angiogenic support, extracellular matrix remodeling, neuroimmune sensitization, and malignant progression. Of the six RTK axes examined, EGFR, VEGFR, and IGF1R have the strongest mechanistic and pharmacological support, whereas PDGFR, FGFR, and MET remain biologically plausible targets. Even though phytochemicals do not consistently act as receptor-selective RTK inhibitors, evidence shows they reduce RTK-centred signaling networks by targeting common downstream hubs. As a result of their effects at the network level, the phytochemicals may help to suppress lesion proliferation, angiogenesis, invasion, fibrosis, inflammation, oxidative stress, and pain sensitization. Endometriosis should be viewed as an RTK-centered network disease rather than a single-pathway disorder. In this context, phytochemicals act as modulators that attenuate signaling hubs, offering a mechanistically aligned strategy for a disease driven by redundancy and microenvironmental crosstalk.

## 1. Introduction

Endometriosis is a chronic inflammatory condition dependent on estrogen and characterized by tissue similar to the endometrium outside the uterine cavity. It affects about 10% of women of reproductive age worldwide and places a heavy burden on patients through pain, fertility issues, reduced quality of life, and healthcare systems [1,2]. Although retrograde menstruation has long been seen as the main cause, it does not explain all cases since many women with retrograde menstruation do not develop the condition [3,4,5]. Current evidence points to a multifactorial cause where lesion initiation and persistence result from a complex, dynamic network involving immune dysfunction, chronic inflammation, progesterone resistance, local estrogen production, oxidative stress, and a permissive peritoneal microenvironment that enables adhesion, invasion, angiogenesis, fibrosis, neurogenesis, and immune evasion [1,3,6,7,8,9].

Sampson’s theory states that viable fragments of endometrium enter the pelvic cavity via retrograde menstruation and then take root at ectopic sites. The double-engines and single-checkpoint theory builds on this model by suggesting that two recurrent factors lead to the development of the disease. Retrograde menstruation serves as the first engine, supplying endometrial progenitor or stem-like cells and simultaneously releasing damage-associated molecular patterns (DAMPs) [10,11], reactive oxygen species (ROS) [12], and other factors that damage tissue and trigger inflammation, potentially leading to genetic changes. Ovulation is the second engine. Follicular fluid during ovulation contains growth factors, soluble extracellular matrix proteins, and estradiol, which may affect endometriotic cells that have refluxed or already exist [4,13]. Consistent with this theory, ovulation and the peritoneal microenvironment actively drive the progression of endometriosis rather than merely providing a permissive site for implantation of refluxed endometrial tissue [13,14,15]. In keeping with this idea, follicular fluid promotes the survival, proliferation, clonogenicity, migration, and invasion of eutopic and ectopic endometrial cells or precancerous cells of the fallopian tubes [5,16,17]. In addition, human peritoneal fluid retains both ovulation- and non-ovulation-derived bioactivities that support cellular survival and transformation-associated phenotypes [18].

The peritoneal fluid also serves as a disease-modifying fluid environment, containing cytokines, chemokines, growth factors, prostaglandins, extracellular matrix components, extracellular vesicles, and substances associated with iron deficiency and oxidative stress [19,20,21,22,23,24,25,26]. Along with defective macrophages, NK cells, neutrophils, dendritic cells, and adaptive immune cells, these factors result in a condition that is both proinflammatory and immunotolerant. This kind of environment hinders the removal of ectopic cells while encouraging adhesion, angiogenesis, invasion, fibrosis, and the persistence of lesions [27,28,29,30,31,32,33,34,35,36,37]. Recent studies of extracellular vesicles also show active communication among ectopic lesions, immune cells, and the peritoneal cavity, thereby further contributing to immune remodeling and disease maintenance [24,38,39]. Overall, these findings support a model in which cyclic ovulation, exposure to follicular fluid, the composition of the peritoneal fluid, immune dysfunction, extracellular vesicle signaling, oxidative stress, and matrix remodeling determine whether refluxed or resident endometrial cells are eliminated, persist, or develop into adhesive, vascularized, and fibrotic lesions.

Previous reviews have primarily focused on intracellular kinases and other druggable signaling pathways involved in endometriosis [9,40,41]. Reviews of plant-derived compounds have generally categorized phytochemicals by chemical class or by their effects on inflammation, oxidative stress, angiogenesis, and cell proliferation [42,43,44,45]. However, these findings have not been systematically compared across receptor tyrosine kinase (RTK) families. The current review concentrates on six representative RTK axes: EGFR, VEGFR, IGF1R, PDGFR, FGFR, and MET. It combines receptor activation with the downstream signaling pathways and the associated disease phenotypes, distinguishing between direct effects on RTKs and indirect modulation of their ligands or downstream signaling cascades. This approach highlights the current evidence for each RTK family and shows how phytochemicals might affect overlapping signaling networks, microenvironmental interactions, and therapeutic responses.

### Literature Search and Narrative Selection

An English-language literature search of PubMed was conducted with no publication date restrictions. The search used combinations and variations of the terms “phytochemicals,” “endometriosis,” “EGFR,” “VEGFR,” “IGF1R,” “PDGFR,” “FGFR,” “MET,” “curcumin,” “resveratrol,” “epigallocatechin gallate,” “quercetin,” “naringenin,” “berberine,” “apigenin,” “luteolin,” “genistein,” and “baicalein.” Additional search terms related to receptor signaling mechanisms, downstream pathways, cellular phenotypes, and therapeutic relevance were also incorporated. Original in vitro, animal, and clinical studies were considered eligible if they investigated RTKs, RTK-associated signaling pathways, or relevant biological phenotypes in endometriosis. Recent review articles were used for citation tracking and contextual comparison. Records retrieved from overlapping searches were deduplicated before title and abstract screening. Because of substantial heterogeneity in experimental models, interventions, and outcome measures, the evidence was synthesized narratively.

## 2. RTK Signaling in Endometriosis: Receptor-Specific Mechanisms and Therapeutic Relevance

The RTK signaling network in endometriosis functions as a highly convergent system, integrating diverse upstream signals into shared downstream modules that promote disease progression. The sections that follow examine these six RTK pathways, focusing on receptor-level mechanisms and their importance for endometriosis therapy. Each receptor—namely EGFR, VEGFR2, IGF1R, PDGFR, FGFR, and MET—has distinct, context-dependent effects in endometriosis, depending on the tissue microenvironment and disease stage. For example, VEGF–VEGFR2 signaling is most directly linked to lesion angiogenesis and neovascular maintenance [46,47], whereas PDGF–PDGFR signaling promotes platelet-induced endothelial- and mesothelial-to-mesenchymal transition, myofibroblast formation, and fibrogenesis [48,49,50]. FGFR2 promotes ectopic endometrial stromal-cell proliferation, migration, and invasion [51], whereas EGFR facilitates EMT and lesion progression through the ERK1–AP-1–MMP-7 axis [52]. IGF1R signaling supports endometriotic graft growth [53] and promotes nerve growth and nociceptive sensitization [54]. HGF–MET signaling enhances endometriotic stromal-cell proliferation, migration, and invasion [55,56]. Moreover, cumulative MET copy-number gain during ovarian clear-cell tumor progression and frequent MET amplification in endometriosis-associated ovarian clear-cell carcinoma implicate this axis in malignant progression in a subset of cases [57,58]. Therefore, a receptor-focused approach is necessary to differentiate well-established drivers from those that are still emerging or only partially characterized. We analyze these six RTK pathways, stressing the receptor-level mechanisms and their therapeutic relevance in endometriosis. A summary of the main findings is given in Table 1.

### 2.1. EGFR

The available evidence indicates that EGFR signaling in endometriosis is more the result of context-dependent activation than of consistent overexpression. EGFR is found in normal, eutopic, and ectopic endometrial tissues, indicating that it is not specific to lesions; nevertheless, the EGF signaling system appears to be dynamically dysregulated in endometriosis [59,60,61]. Functionally, EGF activates EGFR-dependent ERK1/2 and PI3K/AKT signaling in eutopic and ovarian endometriotic stromal cells, thereby upregulating HAS2–hyaluronan signaling and promoting cell migration and invasion [62]. In ovarian endometriosis, EGFR-mediated activation of ERK1 stimulates the AP-1 transcription factor, leading to upregulation of *MMP-7* and induction of epithelial–mesenchymal transition (EMT)-associated phenotypes [52]. It should be noted that pharmacological modulation of this pathway has been shown to occur, as melatonin inhibits EGFR phosphorylation and subsequent PI3K/AKT signaling [63], thus supporting the idea that EGFR could be a potential non-hormonal therapeutic target. However, genetic association studies have yielded inconsistent results, and the value of EGFR as a diagnostic or prognostic biomarker remains uncertain [64,97,98].

### 2.2. VEGFR

Accumulating evidence identifies VEGFR signaling as the main RTK pathway responsible for inducing angiogenesis in endometriosis. The ability of ectopic lesions to survive and persist depends largely on the development of a functional blood supply, which means that the VEGF–VEGFR system is essential for both the development and ongoing maintenance of the lesions [65,66]. Among VEGF family members, VEGF-A is a key pro-angiogenic factor and is consistently elevated within the peritoneal microenvironment of endometriosis [67]. The expression of VEGF is further increased by inflammatory mediators such as TGF-β1 in the surrounding stromal and immune cells. Moreover, both VEGF and its main receptor, VEGFR2 (Flk-1/KDR), are markedly upregulated in endometriotic lesions as compared to eutopic or normal endometrial tissues, and genetic studies involving KDR polymorphisms provide additional support for the functional importance of this receptor [68,69]. Mechanistically, cell-to-cell communication between stromal and endothelial cells—especially through the VEGFA–VEGFR2–FAK signaling pathway—promotes endothelial activation, migration, and the formation of new blood vessels in the lesion microenvironment [70]. Significantly, interventions targeting this pathway have yielded encouraging results: natural compounds such as EGCG and its derivative, Pro-EGCG, inhibit VEGFR2-mediated angiogenesis. At the same time, more general strategies that inhibit VEGF/VEGFR signaling consistently reduce lesion size and vascularization in preclinical models [71,72,73,74,75]. Together, these findings confirm that VEGFR signaling plays a central and therapeutically targetable role in the angiogenesis that occurs in endometriosis.

### 2.3. IGF1R

The IGF1R signaling axis contributes to the pathophysiology of endometriosis by promoting lesion growth, enhancing local estrogen biosynthesis, and facilitating pain sensitization. Clinical and experimental evidence indicates that women with endometriosis exhibit elevated levels of IGF-I in peritoneal fluid, with further enrichment observed in peritoneal fluid mononuclear cells and ectopic stromal cells, reflecting an IGF-rich microenvironment within lesions [76,77]. Mechanistically, IGF-I activates IGF1R/PI3K/AKT signaling in endometriotic stromal cells, promoting c-Jun and CREB recruitment to the ESR2 and CYP19A1 promoters and thereby increasing ERβ expression and aromatase activity. In vivo, IGF1R inhibition attenuated IGF-I-induced graft growth and reduced ERβ and aromatase expression [53]. Beyond proliferative effects, IGF1R signaling also contributes to neurogenic and pain-related processes: macrophage-derived IGF-1 has been shown to promote neurogenesis and sensory hypersensitivity. At the same time, treatment with the IGF1R inhibitor linsitinib attenuates pain-related behaviors in murine models of endometriosis [54]. Collectively, these findings support IGF1R as an active and therapeutically relevant RTK pathway that links hormonal regulation, lesion progression, and pain mechanisms in endometriosis.

### 2.4. PDGFR

The PDGFR axis is less extensively characterized in endometriosis than the EGFR, VEGFR2, and IGF1R axes, with available evidence suggesting its involvement in stromal activation, angiogenesis, and fibrotic remodeling. PDGF signaling promotes endometriotic stromal cell proliferation, migration, and invasion and has been associated with activation of the ERK1/2 pathway and increased expression of VEGF and matrix metalloproteinase-2 (MMP-2) [79,80,81]. In addition, PDGF may contribute to lesion vascularization and fibrosis, particularly within platelet-enriched inflammatory microenvironments, where it supports wound-healing-like processes and extracellular matrix deposition [48,82,83,84]. Despite these mechanistic insights, PDGFR remains only partially validated as a receptor-specific therapeutic target in endometriosis. Preclinical studies investigating PDGFR inhibition, including with imatinib, have yielded preliminary and inconclusive results, highlighting the need for further target-specific and translational validation [85,86,87].

### 2.5. FGFR

The available evidence indicates that FGFR signaling in endometriosis primarily functions as an estrogen-responsive pathway in stromal and neurovascular tissues, rather than as a system characterized by consistently elevated soluble growth factors. While the concentrations of basic fibroblast growth factor (bFGF/FGF2) in peritoneal fluid vary and are not uniformly increased [88], studies based on the lesions show that FGF-9 is present in the ectopic endometrial tissue and is induced by estradiol. In parallel, estradiol induces expression of the FGFR2IIIc and FGFR3IIIc isoforms in endometriotic stromal cells, indicating the presence of an estrogen-responsive FGF-9/FGFR signaling axis [89]. Mechanistically, prostaglandin E2 (PGE2) enhances FGF-9 expression via the EP3–PKCδ–ERK1/2–Elk-1 pathway, thereby promoting stromal cell proliferation [90]. In addition to its effects on cell proliferation, FGFR signaling has also been associated with vascular remodeling in the lesion microenvironment [91]. New evidence also connects FGFR activity to the pathophysiology of pain. In ovarian endometriosis, there is an increase in the number of FGF2-positive mast cells, FGF2 in the ascitic fluid, and lesional FGFR1, and pharmacological inhibition of FGFR1 reduces neurite outgrowth and relieves pain hypersensitivity in vivo [92]. Notably, the FGFR inhibitor AZD4547 has been shown to reduce lesion size in experimental models, supporting the idea that FGFR signaling could be a promising non-hormonal therapeutic target for endometriosis [93].

### 2.6. MET

In endometriosis, an HGF-rich local microenvironment activates MET signaling, thereby promoting stromal-cell proliferation, migration, and invasion [55]. Peritoneal fluid HGF concentrations are elevated in women with endometriosis and positively correlate with revised ASRM disease scores [99]. At the same time, ectopic stromal cells express c-MET, supporting the presence of an HGF-responsive lesional niche [55]. Increased expression of both HGF and c-MET has also been reported in the eutopic endometrium of affected individuals, particularly in cases with highly active disease [95]. Notably, ovarian steroids enhance HGF production by peritoneal macrophages, linking endocrine and inflammatory cues to activation of the HGF/MET pathway [96]. Functionally, HGF promotes stromal cell proliferation and invasiveness, in part by activating proteolytic systems, including the urokinase-type plasminogen activator pathway [55]. More recent evidence further demonstrates that omentum-derived HGF enhances migration of primary endometriotic cells, an effect that can be attenuated by c-MET inhibition [56]. Collectively, these findings suggest that MET represents a promising, albeit less extensively validated, therapeutic target in endometriosis compared with EGFR, VEGFR2, or IGF1R, and is of additional relevance to endometriosis-associated malignant transformation [58].

## 3. Phytochemicals Targeting Molecular Mechanisms in Endometriosis

Current evidence indicates that plant-derived compounds modulate multiple pathogenic processes in endometriosis, including inflammation, oxidative stress, cell proliferation, apoptosis resistance, angiogenesis, invasion, adhesion, and estrogen-dependent signaling [42,100,101,102]. This multitarget profile is particularly relevant to endometriosis, in which pathway convergence and microenvironmental crosstalk may limit the efficacy of narrowly targeted therapies. Accordingly, phytochemicals have emerged as promising candidates for mechanism-based, non-hormonal intervention in lesion development and progression [42,103]. In the following sections, representative phytochemicals are reviewed according to the molecular pathways and pathogenic processes they regulate, with their major mechanisms summarized in Table 2. The evidence was synthesized narratively because the included studies differed substantially in their experimental models, assays, and molecular endpoints. For each phytochemical, we examined whether the reported effects involved direct RTK binding, receptor kinase activity, receptor phosphorylation or expression, ligand availability, or shared downstream signaling. Changes in NF-κB, MMPs, oxidative stress, cytokines, apoptosis, or other disease phenotypes were not considered independent evidence of RTK regulation. When receptor-level validation was unavailable, the findings were interpreted as indirect modulation of the RTK network rather than direct receptor inhibition. The results are summarized in Table 3.

### 3.1. Curcumin

Curcumin is among the most extensively studied phytochemicals in preclinical endometriosis research [169,170]. Experimental evidence supports its anti-inflammatory [104,171], anti-angiogenic [108,109], antiproliferative [109,111,172], and matrix-remodeling effects through regulation of MMP-2, MMP-3, and MMP-9 [105,106,110]. In human endometriotic stromal cells, curcumin suppresses TNF-α-induced NF-κB activation and reduces the expression of key inflammatory and adhesion-related mediators, including IL-6, IL-8, MCP-1, ICAM-1, and VCAM-1, indicating direct modulation of inflammatory signaling and cell–cell interaction pathways [104,170,173]. In experimental models, curcumin promotes lesion regression and attenuates NF-κB nuclear translocation, MMP-3 and MMP-9 expression, and overall inflammatory activity [105,106,107]. In addition, curcumin exerts anti-angiogenic effects by downregulating VEGF expression and reducing microvessel density, and impairs stromal cell survival by inducing G1-phase cell-cycle arrest and apoptosis [108,109]. Its role in tissue remodeling is further supported by inhibition of matrix metalloproteinases, including MMP-2, MMP-3, and MMP-9, and emerging evidence suggests that curcumin may also suppress local estradiol production in endometriotic cells [105,106,110,111]. Collectively, these findings highlight curcumin as a multitarget agent with robust anti-inflammatory and anti-angiogenic properties in endometriosis. However, lesion-specific mechanisms remain incompletely defined, and current clinical evidence for pain relief and therapeutic efficacy remains limited and inconsistent [112,113,130].

Apart from its possible use as a monotherapy, curcumin might also act as an adjunctive sensitizer. Because curcumin suppresses PI3K, AKT, ERK, and NF-κB phosphorylation in endometrial stromal cells from women with endometriosis [174], it may attenuate survival signaling implicated in progesterone resistance [175]. Clinical evidence is currently limited to a randomized trial showing that adjunctive nanocurcumin improved pain, quality of life, and sexual function when added to the progestin dienogest, although its effects on endometrioma size were inconsistent [113]. Whether curcumin can enhance the efficacy of GnRH agonists or antagonists, aromatase inhibitors, RTK or PI3K/mTOR inhibitors, antiangiogenic agents, or anti-inflammatory drugs in endometriosis remains to be determined [176]. A recent cancer-focused review described curcumin as a potential molecular synergy amplifier and proposed that effective combinations may depend on mechanistic complementarity, suppression of adaptive resistance, and pharmacokinetic synchronization [177]. However, these principles have not been directly validated in endometriosis. Evidence demonstrating that curcumin enhances the efficacy of these agents in endometriotic cells, animal models, or patients remains unavailable. Therefore, such combinations should be considered testable pharmacological hypotheses rather than established synergistic treatments. Future studies should incorporate clinically relevant exposures, formal synergy analyses, and pharmacodynamic target-engagement assessments.

### 3.2. Resveratrol

Resveratrol has been extensively investigated as a multitarget phytochemical in endometriosis, with evidence supporting its anti-inflammatory, anti-invasive, anti-angiogenic, and antioxidant properties. In experimental models, resveratrol reduces lesion size and suppresses the invasiveness of endometrial stromal cells, indicating direct anti-lesional activity [114]. At the molecular level, it attenuates inflammatory signaling in endometriotic stromal cells, including TNF-α-mediated responses and the expression of key cytokines and chemokines such as MCP-1, IL-6, IL-8, and RANTES, potentially through SIRT1-dependent mechanisms [116,117]. Resveratrol may also modulate local estrogenic activity, as coadministration with oral contraceptives has been shown to reduce aromatase and COX-2 expression in the eutopic endometrium [115]. In addition, it exerts anti-angiogenic and anti-remodeling effects by downregulating VEGF, TGF-β, MMP-2, and MMP-9, with clinical evidence from an exploratory randomized study supporting its impact on matrix remodeling pathways [119,120]. In preclinical models, resveratrol further reduces oxidative stress and lipid peroxidation. It enhances the sensitivity of endometriotic stromal cells to TRAIL-mediated apoptosis by downregulating survivin, rather than directly inducing apoptosis [118,121]. Despite these promising mechanistic findings, clinical evidence remains inconsistent, particularly regarding pain relief, as randomized placebo-controlled trials have not demonstrated significant superiority over placebo. Collectively, resveratrol represents a biologically active, multitarget compound with potential therapeutic relevance, although its clinical efficacy in endometriosis requires further validation [122].

### 3.3. Epigallocatechin Gallate (EGCG)

EGCG is among the most well-supported anti-angiogenic phytochemicals in endometriosis. Current evidence indicates that its primary therapeutic effect is mediated through inhibition of vascular development, with comparatively modest contributions from matrix remodeling pathways. In experimental models, EGCG reduces lesion size and suppresses VEGF-C/VEGFR2 signaling, supporting a central role in anti-angiogenic regulation [71,123]. Advanced delivery strategies, including EGCG-loaded nanoparticles, further enhance its therapeutic efficacy by reducing matrix metalloproteinase activity, oxidative stress, angiogenesis, and overall lesion burden, suggesting that formulation optimization may improve clinical potential [124]. At the molecular level, EGCG inhibits MMP-2 activity and suppresses MT1-MMP-dependent activation of MMP-2, providing mechanistic support for its secondary effects on extracellular matrix remodeling [125,126]. Collectively, these findings position EGCG as a predominantly anti-angiogenic agent in endometriosis, with MMP inhibition functioning as a complementary rather than primary mechanism.

### 3.4. Quercetin

Quercetin has been primarily characterized by its antiproliferative and pro-apoptotic effects in endometriosis-related models, with comparatively limited direct evidence supporting its anti-inflammatory activity. In immortalized endometriosis-associated epithelial cell lines, quercetin inhibits cellular proliferation, induces cell-cycle arrest, disrupts mitochondrial membrane potential, increases oxidative stress, and promotes apoptosis, accompanied by suppression of AKT- and MAPK-dependent signaling pathways [127]. In vivo studies further demonstrate that quercetin reduces lesion size in rodent models, modulates endocrine-related parameters, lowers circulating TNF-α levels, and improves systemic redox balance [128,129]. Although direct lesion-specific evidence for COX-2/PGE2 inhibition remains limited, its anti-inflammatory potential is biologically plausible, as quercetin has been shown in other systems to suppress COX-2/PGE2- and TNF-α/NF-κB-mediated inflammatory signaling [130,131,132]. Overall, current evidence suggests that quercetin acts predominantly by inhibiting cell growth and inducing apoptosis, while its anti-inflammatory effects in endometriosis remain less well established and warrant further investigation.

### 3.5. Naringenin

Naringenin has demonstrated anti-endometriotic potential primarily through antiproliferative, pro-apoptotic, anti-invasive, and antioxidant mechanisms. In human endometriosis cell models, naringenin inhibits cellular proliferation and induces mitochondria-mediated apoptosis, characterized by mitochondrial depolarization, increased ROS generation, and modulation of AKT- and MAPK-dependent signaling pathways [133]. In vivo, naringenin reduces lesion progression in rodent models, accompanied by decreased expression of proliferation- and signaling-related markers, including VEGF, PCNA, TAK1, and PAK1. Mechanistically, it also activates the Nrf2/Keap1 antioxidant pathway, leading to upregulation of cytoprotective enzymes such as HO-1 and NQO1, while suppressing matrix metalloproteinases MMP-2 and MMP-9, thereby supporting its anti-invasive properties [134]. In contrast, its effects on endocrine regulation remain uncertain. Although naringenin inhibits aromatase activity in cell-free systems, it does not significantly reduce aromatase activity in cultured human endometrial stromal cells, leaving its role in modulating local estrogen production unresolved [135]. Although naringenin suppresses TGF-β1-induced cardiac fibroblast activation and collagen I/III synthesis in a non-gynecologic model [178], its antifibrotic effects in endometriosis have not been directly demonstrated. In endometriosis models, current evidence supports mitochondria- and endoplasmic reticulum stress-mediated apoptosis through MAPK/AKT modulation [133], regulation of Nrf2/Keap1/HO-1 redox signaling, and inhibition of invasion through MMP-2/9 downregulation [134]. Its effects on endocrine signaling and endometriosis-associated fibrosis remain to be established.

### 3.6. Berberine (BBR)

Berberine has demonstrated antiproliferative and anti-invasive effects in endometriosis-related stromal cell models, although direct in vivo evidence specific to endometriosis remains limited. In human endometrial stromal cells, berberine inhibits proliferation, colony formation, migration, and invasion, accompanied by downregulation of matrix metalloproteinases, including MMP2, MMP4, and MMP9, at least partly through suppression of miR-429 [137]. In LPS-stimulated ectopic stromal cells derived from adenomyosis, berberine further reduces the expression of pro-inflammatory and pro-invasive mediators, including IL-6, IL-8, TGF-β, VEGF, and MMP2, while promoting apoptosis and cell-cycle arrest via modulation of TLR4-associated inflammatory signaling, providing indirect support for its anti-inflammatory and anti-invasive potential in ectopic gynecologic stromal disorders [138]. In vivo evidence remains more robust for protoberberine-rich fractions than for purified berberine, as demonstrated by a metabolomics-guided rat study that prevented recurrence of endometriosis [139]. Additionally, berberine’s broader antioxidant properties may be relevant to disease pathophysiology, although direct redox-related evidence in endometriosis is still limited [179]. Berberine is a promising therapeutic candidate for endometriosis, although further studies are needed to validate its efficacy and clarify its specific mechanisms of action.

### 3.7. Apigenin

Apigenin exerts antiproliferative, pro-apoptotic, and anti-inflammatory effects in endometriosis-related in vitro models. In primary endometriotic stromal cells, it suppresses TNF-α- induced proliferation and COX-2/PGE_2_ synthesis via NF-κB inactivation [141]. In VK2/E6E7 and End1/E6E7 epithelial cell lines, apigenin induces cell-cycle arrest and apoptosis, accompanied by mitochondrial depolarization, elevated cytosolic Ca^2+^, ROS accumulation, lipid peroxidation, and activation of ER stress and the unfolded protein response. These apoptotic effects involve ERK1/2, JNK, and AKT signaling [140]. In primary endometriotic stromal cells, apigenin further attenuates TNF-α-induced proliferation and inflammatory responses, including prostaglandin E2 production, by inhibiting NF-κB signaling [141]. Collectively, current evidence supports apigenin as a predominantly anti-mitogenic and apoptosis-promoting compound, with additional NF-κB-mediated anti-inflammatory effects in endometriosis.

### 3.8. Luteolin

Luteolin exerts multi-level anti-endometriotic effects through both cell-intrinsic and immune-modulatory mechanisms. In VK2/E6E7 and End1/E6E7 epithelial cell lines used as endometriosis-related models, luteolin inhibits proliferation through cell-cycle arrest. It induces apoptosis, accompanied by dose-dependent increases in cytosolic Ca^2+^, ROS production, and lipid peroxidation [142]. These effects are associated with inhibition of ERK1/2, JNK, and PI3K/AKT signaling, as well as activation of p38 MAPK [142,180]. In vivo, intraperitoneal administration of luteolin reduces lesion size in murine models, supporting its anti-lesional efficacy. In 12Z human endometriotic cells, luteolin further activates caspases-3, -8, and -9, reinforcing its pro-apoptotic activity [142,143]. Beyond direct effects on lesion cells, luteolin also modulates the immune microenvironment by suppressing the expression of chemokines CCL2 and CCL5 and inhibiting alternative macrophage activation induced by endometriotic cell-conditioned media, thereby reducing the pro-lesional M2-like macrophage phenotype [143]. Together, these findings indicate that luteolin reduces proliferative capacity, enhances apoptotic susceptibility, and attenuates macrophage-mediated support within the endometriotic microenvironment.

### 3.9. Genistein

Genistein should be interpreted with caution in the context of endometriosis, as its biological effects appear to be highly dependent on dose, route of administration, and physiological context. In murine models of peritoneal endometriosis, genistein has been shown to downregulate estrogen receptor-α (ERα) and upregulate ERβ expression, along with reductions in pro-inflammatory and pro-angiogenic mediators, including TNF-α, IL-6, VEGF, and HIF-1α, thereby supporting combined endocrine-modulatory, anti-inflammatory, and anti-angiogenic effects [144,164]. Additional studies have reported increased levels of antioxidant enzymes, such as superoxide dismutase (SOD) and glutathione peroxidase (GPx), in peritoneal fluid, suggesting a potential role in mitigating oxidative stress [145]. However, earlier evidence from rat models demonstrated that pharmacological administration of genistein, in contrast to physiologically relevant dietary exposure, accelerated the progression of surgically induced endometriosis, highlighting the critical influence of bioavailability and route of exposure on therapeutic outcomes [146]. Collectively, these findings suggest that genistein exhibits context-dependent anti-endometriotic activity and should be considered a conditional rather than uniformly protective compound, with its efficacy determined by dosing strategy and biological context.

### 3.10. Baicalein

Baicalein has emerged as a promising phytochemical in endometriosis, exhibiting antiproliferative, anti-inflammatory, and microenvironment-modulating activities. In primary stromal cells derived from ovarian endometriosis, baicalein reduces cell viability, induces G0/G1 cell-cycle arrest, and downregulates key proliferation- and survival-related proteins, including Bcl-2, PCNA, and cyclin D1, through an NF-κB-dependent mechanism [147]. More recent studies employing immortalized patient-derived ovarian endometriotic stromal cells and autologous transplant mouse models further demonstrate that baicalein suppresses lesion progression, decreases pro-inflammatory cytokine expression, and induces mitochondrial dysfunction characterized by increased calcium flux, mitochondrial depolarization, and ROS generation, and inhibits MAPK and PI3K/AKT signaling pathways [148]. Beyond direct effects on stromal cells, baicalein also modulates the lesion microenvironment by reversing iron overload-associated macrophage ferroptosis, restoring phagocytic function, and upregulating GPX4 expression in vitro [149]. Collectively, these findings position baicalein as a multitarget agent that simultaneously disrupts stromal cell survival pathways and ameliorates ferroptosis-linked immune dysfunction in endometriosis.

## 4. Discussion

### 4.1. Compartment-Specific RTK Signaling Across Endometriosis Development and Lesion Progression

Endometriosis may be biologically primed before an ectopic lesion becomes clinically visible. The eutopic endometrium of affected women is not merely normal tissue awaiting displacement; several studies suggest that it already exhibits altered programs of proliferation, apoptosis resistance, adhesion, invasion, and growth-factor signaling [181,182,183]. This may help explain why only a fraction of refluxed endometrial fragments can persist in the peritoneal cavity and develop into lesions [181,183]. In this context, the EGF/ErbB system provides a useful example of compartment-specific RTK regulation. Ejskjaer et al. reported higher HER1–3 mRNA expression in eutopic endometrium from women with endometriosis than in healthy endometrium, whereas ovarian endometriomas showed lower HER1–3 expression and no detectable HER4 expression [61]. Earlier work also found lower EGFR mRNA expression in endometriotic implants and endometrioma cyst walls than in paired eutopic endometrium [184]. These findings argue against a simple “same tissue in the wrong place” model. A more realistic view is that eutopic endometrium, superficial peritoneal lesions, and ovarian endometriomas represent related but biologically reprogrammed compartments, with different RTK-related signaling states shaped by their local microenvironments [185,186,187].

Among peritoneal lesions, red lesions appear to represent a more biologically active phenotype than black, white, or fibrotic lesions [95,188]. Khan et al. reported that HGF and its receptor c-MET were significantly increased in the eutopic endometrium of women with endometriosis. At the same time, red peritoneal lesions showed stronger immunoexpression of HGF, c-MET, VEGF, PCNA, and higher microvessel density than other lesion types [95]. Notably, these tissue-based markers did not show a clear r-ASRM stage-dependent pattern, suggesting that local lesion activity may be better reflected by lesion morphology and microenvironmental signaling than by anatomical stage alone [95]. This interpretation is supported by later work showing that women with dominant red peritoneal lesions had higher serum and peritoneal fluid HGF levels than those with other pigmented lesions or ovarian chocolate cysts, without a major difference between stage I–II and stage III–IV disease [189]. Functionally, the HGF/c-MET system can promote proliferation and invasion of endometrial and endometriotic stromal cells through autocrine and paracrine pathways [55]. Therefore, HGF/c-MET signaling should be described less as a simple stage-dependent marker and more as an activity-linked RTK axis in peritoneal endometriosis [55,95,188,189]. However, soluble c-Met may show stage-associated changes in serum and peritoneal fluid, suggesting that tissue expression and soluble receptor measurements should not be interpreted as identical biological readouts. [190].

As the disease extends from superficial peritoneal implants to ovarian endometrioma and more anatomically advanced disease, the dominant RTK-related signals appear to shift from early lesion survival and angiogenesis toward stromal remodeling, matrix reorganization, fibrosis, and pain-associated tissue remodeling [84,191]. VEGF/VEGFR signaling remains central to this process. Endometriotic lesions with high proliferative activity showed higher microvessel density and stronger VEGFR2 expression, and were also associated with higher serum and peritoneal fluid VEGF-A levels [192]. This supports the view that VEGFR2 is more closely linked to angiogenic and proliferative lesion activity than to anatomical stage alone [192,193]. In ovarian endometriosis, PDGFRA was reported to be significantly higher than in paired eutopic endometrium, suggesting that PDGF/PDGFR signaling may participate in the stromal-remodeling phenotype of ovarian lesions [194]. This interpretation is biologically plausible because PDGF can stimulate endometrial stromal cell proliferation, motility, and contractility, and earlier work also suggested a role for PDGF in the maintenance or proliferation of endometriotic stromal cells [79,80]. However, the fibrotic character of endometrioma walls should not be attributed solely to PDGFRA. Direct evidence for endometrioma-associated fibrosis is stronger for TGF-β1/Smad signaling, as endometrioma cyst walls show increased fibrotic markers and TGF-β1 expression, with TGF-β1 promoting fibrosis in endometrioma-derived stromal cells [136]. Therefore, PDGFRA is best described as a stromal-remodeling RTK candidate in ovarian endometriosis. At the same time, fibrosis in endometrioma is likely driven by a broader wound-healing network that includes TGF-β, platelets, macrophages, myofibroblasts, extracellular matrix remodeling, and possibly PDGF-related signaling [84,136,191].

Other RTK-related pathways add further layers to this lesion-type-based disease model. FGFR2 is highly expressed in ectopic endometriotic tissues compared with paired eutopic endometrium and normal endometrium [51]. Functionally, FGFR2 silencing suppresses ectopic stromal cell viability, proliferation, migration, invasion, and TGF-β1-induced EMT-like changes, with ERK signaling acting as a downstream pathway [51]. The IGF axis also supports the idea that peritoneal lesions, ovarian endometriomas, and deep lesions are not identical biological compartments. IGF-I and IGF-II are differentially expressed in eutopic and ectopic endometrium, suggesting that local IGF signaling may contribute to autocrine or paracrine growth regulation in endometriotic lesions [195]. IGF-1 isoforms are expressed in stromal cells of both eutopic and ectopic endometrium, but their expression differs between red peritoneal lesions and ovarian endometriotic cysts [196]. More recent data also suggest differential IGF-1 isoform expression among DIE lesions, ovarian endometriomas, and eutopic endometrium, supporting the view that ovarian endometrioma alone and DIE-associated ovarian disease may follow partly different molecular programs [197]. Therefore, FGFR2 can be described as a functional RTK driver of ectopic stromal cell growth and invasiveness. In contrast, the IGF system is better framed as an IGF/IGF1R-related growth-factor axis that helps distinguish lesion niches rather than as direct evidence of uniform RTK receptor upregulation across all endometriotic tissues [51,195,197]. Taken together, the available literature does not support a simple model in which RTK receptor expression increases linearly from stage I to stage IV. A more defensible model is spatial- and lesion-type-based. The eutopic endometrium of affected women appears to harbor a preconditioned growth-factor program. In contrast, early active peritoneal lesions preferentially involve HGF/c-MET, EGF/ErbB, and VEGF/VEGFR-related signaling to support lesion survival, proliferation, angiogenesis, and local tissue interactions [61,95,181,189,192]. Ovarian endometriomas show additional stromal-remodeling and growth-factor features, including PDGFRA-, FGFR2-, and IGF-axis alterations, although these pathways should not be interpreted as direct evidence of a uniform RTK increase across all lesion types [51,194,195,196,197].

Emerging RTK Axes and Lesion-Specific Signaling, including the Gas6/AXL axis, TrkA, TrkB/NTRK2, and additional ErbB receptors, remains limited and is often confined to specific lesion types or disease phenotypes. Direct evidence of receptor activation is also incomplete. These receptors are therefore discussed separately as emerging RTK axes. This distinction reflects the maturity of the available evidence rather than the biological importance of these pathways, as they may still contribute to lesion survival, immune regulation, neurogenesis, and pain sensitization. Neurotrophin-related RTKs illustrate this point. NGF/TrkA is strongly expressed in painful deep adenomyotic nodules and has been associated with deep dyspareunia and increased nerve bundle density in posterior-compartment endometriosis [198,199]. BDNF/TrkB expression is higher in ovarian endometriotic lesions than in eutopic and normal endometrium, and eutopic BDNF/TrkB expression has been correlated with the severity of dysmenorrhea [200]. NTRK2 inhibition can induce death of endometriotic stromal cells and lesion regression in experimental models, supporting BDNF/NTRK2 as a lesion-maintenance pathway rather than merely a pain marker [201]. More recent work further suggests that NGF-TrkA signaling may be more directly involved in endometriosis-associated pain than BDNF-TrkB or VEGFR1 signaling [202]. Thus, RTK signaling in endometriosis is better described as a compartment-specific activation program than as a simple stage-dependent marker: active peritoneal lesions emphasize survival and angiogenesis, ovarian endometriomas add stromal and growth-factor remodeling, and deep or pain-associated lesions recruit stronger neurogenic RTK-related signaling.

### 4.2. Phytochemicals as RTK-Network Attenuators in Endometriosis

**Figure 1:**
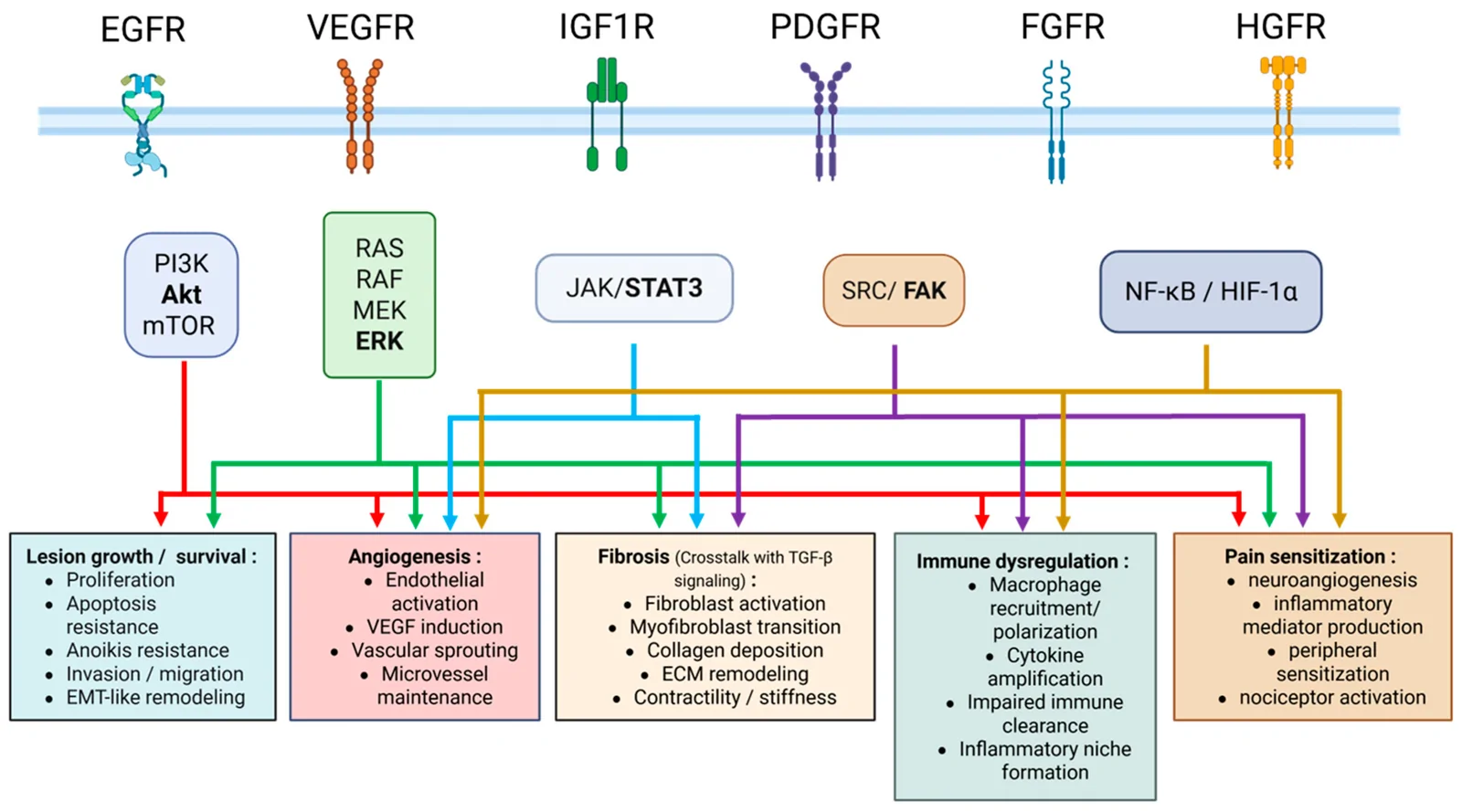
Convergent RTK signaling networks driving major pathogenic phenotypes in endometriosis. In the endometriotic microenvironment, enrichment of growth factors activates multiple RTK axis, including EGFR, VEGFR2, IGF1R, PDGFR, FGFR, and c-MET. These upstream signals converge on shared intracellular signaling hubs, most prominently the PI3K–AKT–mTOR, RAS–RAF–MEK–ERK, JAK/STAT3, and SRC/FAK pathways, with additional transcriptional crosstalk mediated by NF-κB and HIF-1α. The integration of these signaling networks drives key disease phenotypes, including lesion growth and survival, angiogenesis, fibrosis, immune dysregulation, and pain sensitization. Each colored line represents the connection between a specific signaling node and its corresponding downstream disease phenotype. The figure illustrates this network-based architecture of RTK signaling in endometriosis, highlighting the central role of pathway convergence and supporting therapeutic strategies that target shared signaling nodes rather than individual receptors.

Current evidence indicates that endometriosis is not driven by a single dominant pathway but by a highly interconnected signaling network in which steroidal, inflammatory, and growth-factor cues converge on shared intracellular hubs, particularly the PI3K–AKT–mTOR and MAPK/ERK axes. Contemporary models of endometriosis pathobiology emphasize pathway convergence and signaling redundancy, suggesting that therapeutic strategies should move beyond single-node inhibition toward network-level modulation (Figure 1) [40,41,203,204]. Multiple RTK systems contribute to this convergence [61]. Dysregulation of the EGF/EGFR axis has been observed in eutopic endometrium, implicating altered EGFR signaling in disease initiation. Genetic and functional studies support a central role for VEGFR2 (KDR) in angiogenesis and lesion maintenance [69]. FGFR1 overexpression in ectopic lesions is associated with increased disease severity and recurrence, while pharmacological inhibition attenuates lesion progression in vivo [93,205,206]. The IGF axis further reinforces proliferative and endocrine signaling through IGF1R/PI3K/AKT activation and upregulation of ERβ and aromatase [53], whereas MET signaling—driven by elevated hepatocyte growth factor (HGF) in peritoneal fluid—promotes stromal-cell proliferation and invasion [55]. Additional RTK-related pathways, including PDGFR-β-mediated fibrogenesis [50] and Gas6–AXL signaling, further highlight the breadth of RTK involvement in shaping the endometriotic microenvironment [207].

Within this framework, phytochemicals are more appropriately conceptualized as RTK-network attenuators rather than single-target inhibitors. Evidence from medicinal chemistry and pharmacology demonstrates that natural compounds can modulate multiple RTK-related pathways either through direct receptor-level interactions or via downstream kinase networks [208,209]. In endometriosis, the most robust evidence centers on angiogenic signaling, particularly the VEGFR2 axis. EGCG suppresses lesion growth and angiogenesis through inhibition of VEGFC/VEGFR2 signaling [71,123], while its derivative Pro-EGCG exhibits enhanced anti-angiogenic efficacy in vivo [72]. Notably, Pro-EGCG also inhibits EGF-driven angiogenesis via the EGF/HIF-1α/VEGF cascade, illustrating functional crosstalk between EGFR and VEGFR pathways [210]. Similarly, ginsenoside Rg3 attenuates angiogenesis through VEGFR2-dependent PI3K/AKT/mTOR signaling, supporting the concept that phytochemicals can target RTK signaling at multiple hierarchical levels [211]. However, an important pharmacological distinction is that flavonoids should not be treated as interchangeable RTK network modulators. Flavonoids should not be viewed as interchangeable RTK-network modulators. Quercetin is a flavonol, apigenin and luteolin are flavones, naringenin is a flavanone, genistein is an isoflavone, and EGCG is a flavan-3-ol. Differences in ring orientation, planarity, hydroxylation, and galloylation affect solubility, membrane permeability, metabolism, and protein interactions. These features influence whether a compound reaches and engages RTK-linked intracellular nodes [212]. Glycosylation further changes solubility, intestinal hydrolysis, transporter use, and absorption. Glycosides and aglycones therefore have distinct metabolic profiles [213]. For example, naringin is converted to naringenin, while absorbed flavonoids undergo glucuronidation, sulfation, and methylation. Methylation may improve membrane transport and metabolic stability but also changes the chemical groups available for protein binding [214,215]. Formulation can also alter systemic exposure and cellular uptake. Human studies show marked formulation-dependent differences in quercetin bioavailability, but greater exposure does not establish stronger RTK affinity or selectivity [216]. Accordingly, placing structurally distinct phytochemicals around the same network nodes should not imply that they are interchangeable multitarget agents. Recent endometriosis studies illustrate this distinction. Quercetin reduces AKT and ERK phosphorylation, indicating modulation of downstream pathways rather than direct RTK binding [217]. Its association with the PDGFRB/MAPK axis is supported by receptor expression and genetic interference data, but direct PDGFRB binding, receptor phosphorylation, and kinase inhibition were not examined [155]. Chemical proteomics also showed that EGCG and ProEGCG bind different non-RTK proteins, MTDH and PXK, respectively, and regulate distinct angiogenic pathways [210]. Thus, similar effects on PI3K/AKT/mTOR, VEGF, MMPs, inflammation, or apoptosis indicate pathway convergence. They do not establish equal potency, bioavailability, receptor engagement, or relevance to endometriosis.

Beyond direct receptor modulation, phytochemicals may attenuate RTK signaling indirectly by reducing ligand availability, an approach particularly relevant in a disease characterized by pathway redundancy. For example, resveratrol decreases the expression of IGF-1 and HGF in endometriotic stromal cells, suggesting downregulation of upstream inputs to IGF1R and MET signaling [152]. In contrast, direct evidence for phytochemical modulation of FGFR and PDGFR pathways in endometriosis remains limited, with current support derived largely from broader kinase-targeting studies rather than disease-specific intervention models [40,41,218]. This distinction underscores an important therapeutic principle: phytochemicals may exert benefit not by fully inhibiting individual RTKs, but by simultaneously dampening proliferative, angiogenic, invasive, and fibrotic signaling across interconnected RTK-dependent networks.

**Figure 2:**
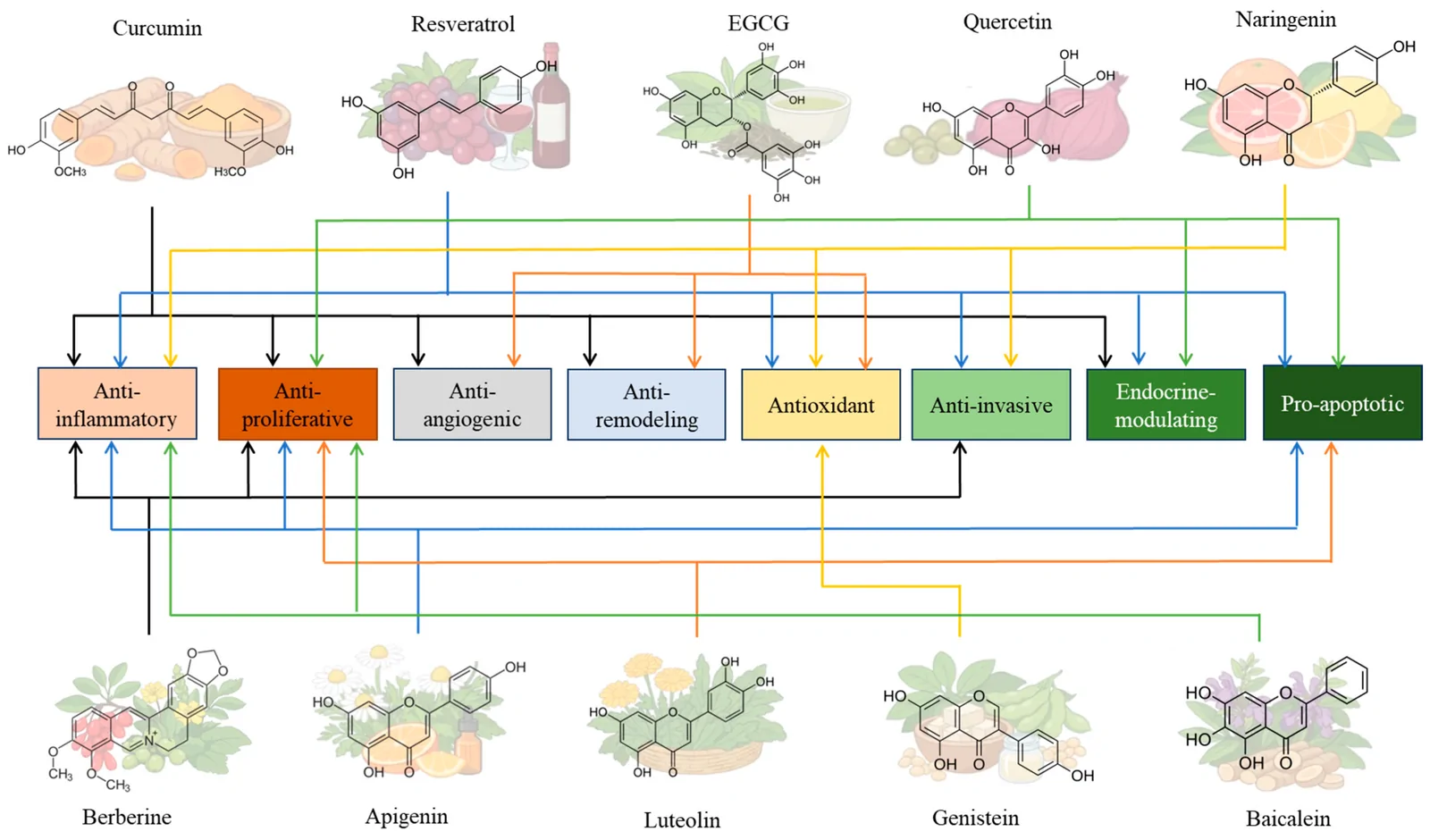
Phytochemicals mapped onto RTK axis and convergent downstream signaling nodes in endometriosis. Phytochemicals investigated in endometriosis and related RTK-driven disease models may attenuate lesion-promoting signaling at multiple levels within this network. Rather than functioning solely as highly selective receptor inhibitors, many act as net-work-level modulators that simultaneously interfere with multiple RTK axes and their downstream signaling pathways. By targeting both receptor-level activation and shared intracellular hubs, these compounds collectively suppress major pathogenic outputs of endometriosis, including cellular proliferation and survival, angiogenesis, extracellular matrix remodeling, immune dysregulation, and pain-related signaling. The colored lines in the upper and lower rows connect each phytochemical to the corresponding pathological phenotypes that it may attenuate. This systems-level mode of action further supports the concept that phytochemicals are best understood as RTK-network attenuators, providing a mechanistically aligned approach to managing a disease characterized by signaling redundancy and microenvironmental complexity.

Such a multitarget, moderate-intensity strategy may be particularly well suited to endometriosis, a chronic benign disease in which complete pathway suppression may be biologically effective but clinically constrained by toxicity. In this context, phytochemicals offer a systems-level approach that aligns with the network-driven nature of disease pathogenesis (Figure 2). Future research should therefore focus on defining the RTK signaling nodes most susceptible to phytochemical modulation, determining pharmacologically achievable exposure levels, and identifying lesion subtypes or molecular contexts in which such interventions are most effective. Overall, phytochemicals are best conceptualized as multitarget network modulators rather than single-pathway agents in endometriosis. However, current evidence remains largely preclinical, and future studies should prioritize receptor-level validation, pharmacokinetic optimization, and translational investigation to define their therapeutic potential more precisely.

### 4.3. Phytochemical Pleiotropy and RTK-Network Modulation

Recent reviews describe phytochemicals as multi-target compounds that act on several processes involved in endometriosis. These processes include inflammation, oxidative stress, angiogenesis, fibrosis, and endocrine signaling [45,176,219,220,221,222]. Curcumin is among the most extensively studied phytochemicals in preclinical endometriosis research [169,170]. In endometriosis-related stromal cells, curcumin suppresses AKT, ERK, and NF-κB phosphorylation and attenuates TNF-α-driven inflammatory signaling [104,171,174]. It also reduces VEGF expression [109] and alleviates oxidative stress by decreasing ROS and MDA levels and increasing SOD activity, thereby inhibiting NLRP3/caspase-1/GSDMD-mediated pyroptosis [223]. Curcumin further reduces estradiol production and endometriotic-cell proliferation [111] and modulates extracellular-matrix turnover by suppressing MMP-2, MMP-3, and MMP-9 [105,106,110]. Although activation of Nrf2/Keap1/HO-1 and inhibition of TGF-β/SMAD signaling have been demonstrated in non-endometriosis models [224,225], these mechanisms and a direct antifibrotic effect have not been established in endometriosis. These broad effects do not mean that curcumin directly inhibits an RTK. AKT, ERK, NF-κB, and VEGF are shared signaling nodes that receive inputs from RTKs, cytokine receptors, steroid receptors, and redox pathways. Changes in these molecules therefore indicate downstream pathway regulation rather than receptor selectivity. Direct RTK regulation requires stronger evidence, such as changes in receptor phosphorylation or kinase activity, physical binding to the target, or genetic or pharmacological rescue. Recent studies support this distinction. Curcumin regulated the oxidative stress–pyroptosis axis in endometriosis [223]. Another study found that curcumin reduced VEGF expression, cell migration, and proliferation. Interestingly, curcumin treatment was associated with increased IGF1 and IGF2 mRNA expression in eutopic endometrial stromal cells from women with endometriosis, although these changes did not reach statistical significance [172]. Thus, changes in ligand mRNA alone cannot establish whether IGF1R is activated or inhibited. Chemical proteomics also identified different direct protein targets for EGCG and ProEGCG [210]. Other studies linked quercetin to AKT/ERK/p53-dependent decidualization and senescence [217] and showed that β-elemene induced ferroptosis and reduced fibrosis through MAPK14/STAT3/GPX4 signaling [226]. The present review therefore maps these phytochemical effects onto the EGFR, VEGFR2, IGF1R, PDGFR, FGFR, and MET networks. It separates direct receptor regulation from downstream signaling changes and phenotype-level effects.

### 4.4. Nutritional Perspective: Therapeutic Potential and Limitations of Phytochemicals

From a nutritional perspective, curcumin, resveratrol, quercetin, genistein, and epigallocatechin gallate are food-derived bioactive compounds with potential adjunctive value in endometriosis. Preclinical studies report antioxidant, anti-inflammatory, antiproliferative, and antiangiogenic effects. Depending on the compound, these effects involve PI3K–AKT, MAPK/ERK, NF-κB, HIF-1α/VEGF, redox, and metabolic pathways [42,43,176,210,227]. These pathways contain shared signaling nodes used by RTKs, cytokine receptors, estrogen receptors, and metabolic regulators. Their modulation therefore does not independently demonstrate direct RTK binding or receptor inhibition. In addition, evidence from ProEGCG should not be extrapolated directly to dietary EGCG, as ProEGCG is a chemically modified prodrug with distinct molecular targets and pharmacokinetic properties [210].

Plant-rich dietary patterns may also influence inflammation, oxidative balance, estrogen metabolism, gastrointestinal symptoms, and the gut microbiome. Current evidence supports evaluating nutrition as an adjunct to multidisciplinary endometriosis management, but it does not establish that a specific diet prevents endometriosis or modifies lesion progression [228,229,230,231]. Habitual dietary intake should also be distinguished from treatment with purified compounds, concentrated extracts, or nanoformulations. These preparations differ in dose, absorption, intestinal and hepatic metabolism, microbiome-dependent biotransformation, and systemic exposure. Phytoestrogens, particularly isoflavones and resveratrol, may also exert estrogenic or antiestrogenic effects depending on dose, metabolites, tissue context, and ERα/ERβ expression [232]. Concentrated catechin-rich preparations require additional caution because green-tea extracts have been associated with dose-dependent hepatotoxicity [233].

Clinical findings remain limited and inconsistent. Oral curcumin at 1 g/day for 8 weeks did not improve pain or quality of life [112]. In contrast, nanocurcumin at 80 mg/day, added to dienogest for 8 weeks, improved pain, quality of life, and sexual function but did not significantly reduce endometrioma size [113]. Resveratrol added to an oral contraceptive did not provide greater pain relief than placebo [122]. These contrasting findings suggest, but do not prove, that formulation and treatment context influence clinical activity. Compound-specific trials remain small, short, and focused mainly on symptoms rather than lesion biology, recurrence, fertility, or long-term safety [231,234]. Phytochemicals should therefore be considered adjunctive dietary or nutraceutical strategies rather than substitutes for established medical or surgical treatments. Standardized formulations, pharmacokinetic and pharmacodynamic analyses, molecular target-engagement assays, and adequately powered trials with lesion-based and reproductive outcomes are required before disease-modifying effects can be established.

### 4.5. Single-Cell, Spatial, and Metabolomic Perspectives

Recent single-cell and spatial studies confirm that endometriotic lesions contain distinct epithelial, stromal, vascular, mesothelial, and immune-cell populations. Single-cell RNA sequencing of ovarian endometriomas identified pro-inflammatory IGFBP5-positive macrophages, exhausted NK cells, and proliferating IQCG-positive and KLF2-positive epithelial populations [235]. However, IGFBP5 expression defines a macrophage state and does not prove IGF1R activation. Combined single-cell and spatial transcriptomics identified separate fibrotic and inflammatory stromal niches in ovarian lesions. The same study found increased WNT5A-associated noncanonical WNT signaling in ectopic stromal cells [236]. Another single-cell atlas detected endometriosis-associated mesothelial cells across ovarian, peritoneal, and deep endometriosis. Ligand–receptor analysis suggested that mesothelial cells may promote stromal progesterone resistance through FN1–AKT signaling [237]. FN1–AKT signaling is primarily associated with integrin and focal adhesion pathways. It does not indicate direct RTK regulation. Spatial transcriptomics of superficial peritoneal lesions also identified epithelial C3 signaling and a pro-repair macrophage state [238]. Integrated single-cell, spatial transcriptomic, and spatial metabolomic analysis of ovarian endometriomas showed enrichment of cell-adhesion, ECM–receptor interaction, and focal-adhesion pathways. It also identified changes in cytochrome P450 activity, lipoprotein particles, and cholesterol metabolism [239]. These pathways provide a metabolic and structural context for RTK signaling but are not specific to RTKs. Another single-cell study linked ovarian iron overload to cellular senescence, altered granulosa-cell differentiation, immune dysfunction, and metabolic reprogramming in endometriosis-associated infertility [240]. Plasma and peritoneal fluid metabolomics further identified changes in phosphatidylcholines, lysophosphatidylcholines, and acylcarnitines [241]. These metabolite changes were modest and were developed mainly as diagnostic markers. The clearest recent RTK-related evidence comes from a large 2026 single-cell atlas of non-hormonally treated eutopic endometrium. This study inferred increased VEGFA–VEGFR1/2 and PGF–VEGFR1 signaling among macrophages, mural cells, and endothelial cells [242]. It provides cell-specific support for VEGFR signaling in endometriosis. However, the analysis was based on transcript expression and predicted ligand–receptor interactions. It did not measure VEGFR phosphorylation or kinase activity. A 2026 multi-ancestry genetic and multi-omics study also linked endometriosis risk to immune regulation, hormonal signaling, inflammation, and tissue remodeling, but did not establish direct RTK activation [243]. Collectively, these studies provide an increasingly detailed cellular, spatial, and metabolic framework for understanding endometriosis and for evaluating the potential actions of phytochemicals. Nevertheless, most investigations characterize the disease at baseline rather than examining responses to phytochemical treatment. Future studies should integrate phytochemical interventions with single-cell and spatial transcriptomics, metabolomics, phosphoproteomics, receptor phosphorylation assays, and target-engagement analyses. Such multimodal approaches would enable identification of the responsive cell populations while distinguishing direct RTK regulation from downstream signaling and phenotype-level effects.

### 4.6. Translational Gaps, Safety Considerations, and Limitations of the Current Evidence

Despite compelling in vitro and animal findings, a substantial gap remains between phytochemical-mediated modulation of RTK-centered signaling and clinical benefit in endometriosis. Many preclinical studies use micromolar concentrations or exposure schedules that may not be achievable within human lesions after oral administration. This concern is especially relevant to curcumin and resveratrol because both compounds have limited systemic bioavailability and undergo rapid metabolism [244,245]. In addition, differences in compound purity, formulation, route of administration, dose, treatment duration, and experimental model complicate direct comparisons across studies. Most investigations also assess downstream signaling pathways or cellular phenotypes without measuring plasma or lesion drug concentrations, receptor phosphorylation, kinase activity, or direct target engagement. Future translational studies should therefore incorporate patient-derived organoids, tissue explants, or primary cells together with clinically relevant exposure levels, pharmacokinetic–pharmacodynamic analyses, receptor-specific biomarkers, and target-engagement assays.

Potential endocrine and long-term safety effects also warrant careful consideration. Isoflavones, resveratrol, and other flavonoids may exert estrogenic or anti-estrogenic effects depending on their concentrations, metabolites, cellular context, and the relative activation of estrogen receptor α (ERα) and β (ERβ) [232,246]. Estrogen receptors exhibit extensive crosstalk with EGFR and IGF1R signaling, while estradiol can increase VEGF expression in endometriotic cells [53,247,248]. Consequently, modulation of RTK-associated pathways may arise, at least in part, through indirect endocrine mechanisms rather than direct RTK inhibition. Clinical evidence likewise remains inconclusive. The addition of resveratrol to an oral contraceptive did not provide additional pain relief, and curcumin monotherapy failed to improve pain or quality of life [112,122]. By contrast, a recent short-term clinical trial reported that curcumin combined with dienogest improved pain, quality of life, and sexual function compared with dienogest alone [113]. Although these findings support further evaluation of combination therapy, they do not establish long-term efficacy or durability of response. Moreover, the effects of prolonged phytochemical use on menstrual cyclicity, ovarian reserve, fertility, pregnancy, and offspring remain insufficiently characterized. Concentrated catechin-rich green tea extracts have also been associated with dose-dependent hepatotoxicity under specific dosing conditions [233]. Accordingly, comprehensive long-term reproductive and organ-specific safety evaluations are essential before widespread clinical application.

The current evidence is further limited by small sample sizes, short follow-up periods, heterogeneous patient populations and lesion types, inconsistent interventions, and nonstandardized outcome measures. Previous systematic reviews have identified confounding factors, incomplete reporting, variable control groups, different outcome definitions, and a limited number of human studies [249,250]. Preclinical models do not fully reproduce the hormonal, immune, vascular, fibrotic, and pain-related features of human endometriosis. Positive publication bias may also overrepresent favorable findings. In addition, mechanistic evidence obtained from cancer or other disease models cannot be directly extrapolated to endometriosis. The present review was restricted to English-language PubMed-indexed literature and used a narrative synthesis. It did not formally pool effect sizes or grade the study-level risk of bias and the certainty of the evidence. The selection of six major RTK axes and representative phytochemicals may also have underrepresented less-studied receptors and compounds. Comparisons across RTK families should therefore be interpreted as qualitative assessments of evidence maturity rather than definitive rankings of therapeutic efficacy.

## 5. Conclusions

In summary, accumulating evidence indicates that endometriosis is governed not by a single dominant pathway but by a redundant, highly interconnected signaling network in which steroidal, inflammatory, angiogenic, and growth factor-mediated inputs converge on shared intracellular hubs, particularly the PI3K–AKT–mTOR and MAPK/ERK pathways. Within this framework, phytochemicals are best conceptualized not as conventional single-target inhibitors, but as network-level attenuators of RTK-driven signaling. By simultaneously modulating multiple interconnected pathways, these compounds may overcome the limitations of narrowly targeted interventions in a disease characterized by signaling redundancy and microenvironmental crosstalk. Importantly, such a multitarget and potentially lower-toxicity strategy may be particularly well suited for the long-term management of chronic benign conditions such as endometriosis.

## Figures and Tables

**Table 1:** Receptor-specific mechanisms and therapeutic relevance of major RTK pathways in endometriosis.

RTK Axis	Core Disease Role	Key Receptor-Specific Mechanism in Endometriosis	Therapeutic Relevance	Key PMIDs
EGFR	Context-dependent lesional signaling driving motility and progression	EGF promotes stromal-cell migration and invasion through EGFR-dependent ERK1/2-AKT activation, HAS2 induction, hyaluronan production, and ERK1–AP1–MMP-7 signaling	EGFR inhibition suppresses downstream signaling and lesion growth; melatonin also attenuates EGFR phosphorylation	[52,59,60,61,62,63,64]
VEGFR2	Angiogenic driver of lesion vascularization and maintenance	Increased VEGF/VEGFR2 expression and VEGFA–VEGFR2–FAK crosstalk support endothelial activation and vascular persistence of ectopic lesions	Anti-VEGF/VEGFR strategies reduce lesion burden; EGCG and Pro-EGCG suppress VEGFR2-related angiogenic signaling	[65,66,67,68,69,70,71,72,73,74,75]
IGF1R	Lesion-promoting, estrogen-amplifying, and pain-sensitizing RTK node	IGF-I activates IGF1R/PI3K/AKT, upregulates ERβ and aromatase, and links macrophage-derived IGF-1 to neuroimmune pain sensitization	IGF1R inhibition suppresses ectopic growth; linsitinib alleviates pain behavior in experimental models	[53,54,76,77,78]
PDGFR	Stromal and microenvironmental activator involved in proliferation, migration, and remodeling.	PDGF stimulates stromal proliferation and motility, with ERK1/2, VEGF, and MMP-2 as major downstream effectors; profibrotic platelet-associated signaling provides indirect support	Imatinib shows partial preclinical inhibitory activity, but receptor-specific validation remains limited.	[48,79,80,81,82,83,84,85,86,87]
FGFR	Estrogen-responsive stromal and neurovascular signaling axis	Estradiol induces FGF-9 with FGFR2IIIc/FGFR3IIIc, while PGE2–FGF-9 signaling promotes stromal proliferation; FGFR1 also contributes to pain-related neurite outgrowth	FGFR inhibition reduces lesion size and pain-associated neural responses in preclinical models	[88,89,90,91,92,93,94]
MET	HGF-driven stromal activation and invasion-promoting signaling	HGF-rich peritoneal microenvironment activates c-Met, enhances stromal proliferation and invasion, and supports migratory behavior of endometriosis cells	c-MET inhibition suppresses migration, but lesion-level therapeutic validation remains less mature	[55,56,58,95,96]

Abbreviations: EGFR, epidermal growth factor receptor; VEGFR2, vascular endothelial growth factor receptor 2; IGF1R, insulin-like growth factor 1 receptor; PDGFR, platelet-derived growth factor receptor; FGFR, fibroblast growth factor receptor; MET, mesenchymal–epithelial transition factor; EMT, epithelial–mesenchymal transition; AP1, activator protein 1.

**Table 2:** Molecular mechanisms, RTK-network associations, and therapeutic relevance of representative phytochemicals in endometriosis.

Compound	Principal Mechanisms in Endometriosis	Major Biologic Effects	Key Pathways/Mediators Affected	Key PMIDs
Curcumin	Anti-inflammatory, anti-angiogenic, anti-proliferative, anti-remodeling, endocrine-modulating	Suppresses inflammatory cytokines, reduces lesion growth and microvessel density, induces cell-cycle arrest and apoptosis, inhibits extracellular matrix remodeling, and may reduce estradiol production	NF-κB, TNF-α, IL-1β, IL-6, IL-8, MCP-1, ICAM-1, VCAM-1, VEGF, MMP-2, MMP-3, MMP-9, E2	[104,105,106,107,108,109,110,111,112,113]
Resveratrol	Anti-inflammatory, anti-invasive, anti-angiogenic, oxidative stress-modulating, endocrine-related	Reduces lesion development and stromal invasiveness, suppresses inflammatory mediators, lowers angiogenic and remodeling factors, improves oxidative stress, and sensitizes cells to apoptosis	SIRT1, TNF-α-related signaling, IL-6, IL-8, MCP-1, RANTES, aromatase, COX-2, VEGF, TGF-β, MMP-2, MMP-9, survivin, TRAIL	[114,115,116,117,118,119,120,121,122]
EGCG	Anti-angiogenic, anti-remodeling, anti-invasive	Inhibits lesion development, suppresses angiogenic signaling, reduces matrix metalloproteinase activity, and attenuates lesion-associated remodeling	VEGFC, VEGFR2, MMP-2, MT1-MMP, PLAU/uPA	[71,123,124,125,126]
Quercetin	Anti-proliferative, pro-apoptotic, potentially anti-inflammatory	Inhibits proliferation, induces cell-cycle arrest and apoptosis, reduces lesion growth, and improves inflammatory/oxidative stress-related parameters	AKT, MAPK, TNF-α, COX-2/PGE2, NF-κB	[127,128,129,130,131,132]
Naringenin	Anti-proliferative, pro-apoptotic, anti-invasive, antioxidant	Suppresses proliferation, induces mitochondrial apoptosis, reduces lesion progression, downregulates prognostic and angiogenic markers, and activates antioxidant defense pathways	AKT, MAPK, VEGF, PCNA, TAK1, PAK1, Nrf2/Keap1, HO-1, NQO1, MMP-2, MMP-9	[133,134,135,136]
Berberine (BBR)	Anti-proliferative, anti-invasive, anti-inflammatory	Inhibits stromal proliferation, colony formation, migration, and invasion; reduces inflammatory and invasive mediators; in vivo evidence is stronger for protoberberine-rich fractions than for purified BBR alone	miR-429, MMP-2, MMP-4, MMP-9, IL-6, IL-8, TGF-β, VEGF, TLR4-linked signaling	[137,138,139]
Apigenin	Anti-proliferative, pro-apoptotic, anti-inflammatory	Induces cell-cycle arrest and apoptosis, increases oxidative and ER stress, and suppresses TNF-α-induced proliferation and prostaglandin production	ERK1/2, JNK, AKT, ROS, ER stress/UPR, NF-κB, PGE2	[140,141]
Luteolin	Anti-proliferative, pro-apoptotic, immune-modulatory	Reduces proliferation and lesion size, activates caspase-dependent apoptosis, suppresses chemokine expression, and inhibits lesion-supportive macrophage polarization	ERK1/2, JNK, PI3K/AKT, p38, caspase-3/-8/-9, CCL2, CCL5	[142,143]
Genistein	Context-dependent endocrine, anti-inflammatory, anti-angiogenic, antioxidant	Under specific dosing conditions, reduces inflammatory and angiogenic markers and increases antioxidant enzymes, although biologic effects vary by route and exposure level.	ER-α, ER-β, TNF-α, IL-6, VEGF, HIF-1α, SOD, GPx	[144,145,146]
Baicalein	Anti-proliferative, anti-inflammatory, mitochondria-mediated cytotoxic, microenvironment-modulating	Reduces stromal viability, induces cell-cycle arrest and cell death, suppresses lesion progression and inflammatory cytokines, and counteracts macrophage ferroptosis-associated dysfunction	NF-κB, Bcl-2, PCNA, cyclin D1, MAPK, PI3K, ROS, mitochondrial calcium flux, GPX4, ferroptosis-related pathways	[147,148,149]

Abbreviations: BBR, berberine; E2, estradiol; EGCG, epigallocatechin gallate; ER, estrogen receptor; ECM, extracellular matrix; GPx, glutathione peroxidase; HO-1, heme oxygenase-1; NQO1, NAD(P)H quinone dehydrogenase 1; ROS, reactive oxygen species; UPR, unfolded protein response.

**Table 3:** Evidence-based classification of RTK modulation by selected phytochemicals relevant to endometriosis.

Phytochemical	Evidence in Endometriosis Models	RTK-Proximal Evidence from Non-Endometriosis Models	Evidence-Based Interpretation
Curcumin	LA: Reduced VEGF expression [109].	KI/RP: Inhibited EGFR kinase activity and reduced EGFR activation/autophosphorylation [150,151]	Endometriosis-specific evidence is limited to ligand regulation; direct receptor inhibition is supported only by contextual models. DB: ND.
Resveratrol	LA: Reduced IGF-1, HGF, and VEGF production or expression [119,152].	DB/RP: Bound the extracellular region of EGFR and reduced EGFR phosphorylation [153].	The only compound in this group with experimentally demonstrated direct RTK binding (DB), although the supporting evidence was generated outside endometriosis models. KI: ND.
EGCG	LA: Reduced VEGFC and VEGFR2 expression/signaling [71]; receptor phosphorylation was not directly demonstrated.	LA/RP: Sequestered VEGF and consequently reduced VEGFR2 activation [154].	Primarily modulates the VEGF–VEGFR axis through regulation of ligand availability (LA) rather than direct receptor binding or receptor kinase inhibition. RTK DB/KI: ND.
Quercetin	Reduced PDGFRβ abundance and downstream RAS–RAF–ERK signaling, without measurements of receptor phosphorylation or kinase activity [155].	KI/RP: Inhibited EGFR kinase activity and phosphorylation [156]. LA/RP: Reduced VEGF secretion and VEGFR2 phosphorylation [157].	Evidence for endometriosis shows receptor abundance/downstream effects, not demonstrated receptor inhibition. RTK-proximal activity is contextual. DB: ND.
Naringenin	LA: Reduced VEGF expression [134].	KI: Inhibited HER2 kinase activity in a biochemical assay [158].	Evidence for endometriosis is limited to ligand regulation. Docking does not establish direct binding. DB/RP: ND.
Berberine	No qualifying DB, KI, RP, or LA evidence; reported effects involved miR-429, MMPs, and cellular phenotypes [137].	KI: Inhibited MET kinase activity [159]. RP: Reduced phosphorylation of EGFR and other RTKs [160].	RTK modulation has not been established in endometriosis; receptor-proximal evidence is derived entirely from contextual models. DB/LA: ND.
Apigenin	DH only: Altered ERK1/2, JNK, and AKT without receptor- or ligand-proximal measurements [140].	RP: Reduced HER2 and IGF1R phosphorylation/autophosphorylation [161,162]. LA: Reduced VEGF expression [163].	Acts through shared downstream hubs in endometriosis models; receptor-proximal effects are supported only by contextual evidence. DB/KI: ND.
Luteolin	LA: Reduced macrophage-associated VEGF [143]. Additional effects involved ERK, JNK, and PI3K–AKT [142].	KI/RP: Inhibited EGFR kinase activity and phosphorylation [156].	Not DH-only, as ligand regulation (LA) was demonstrated in an endometriosis-related model, whereas direct receptor effects remain contextual. DB: ND.
Genistein	LA: Reduced VEGF-A expression [164]; docking alone does not establish direct binding.	KI/RP: Inhibited protein tyrosine kinase/EGFR kinase activity and EGF-induced receptor phosphorylation [165,166].	Endometriosis-specific evidence supports ligand regulation (LA), whereas evidence for direct receptor kinase inhibition is derived from broader contextual models rather than endometriosis-specific studies. DB: ND.
Baicalein	DH only: Suppressed MAPK/PI3K signaling without measuring a named RTK, receptor phosphorylation, or ligand availability [148].	RP: Reduced EGFR phosphorylation/autophosphorylation [167,168].	Acts through shared downstream hubs in endometriosis models; EGFR-proximal evidence is contextual. DB/KI/LA: ND.

Abbreviations and evidence criteria: DB, experimentally demonstrated direct physical binding to an RTK; KI, inhibition of receptor kinase activity demonstrated in a biochemical kinase assay; RP, reduction in phosphorylation or autophosphorylation of a specified RTK; LA, alteration of cognate RTK ligand expression, secretion, extracellular availability, or sequestration; DH, modulation limited to shared downstream signaling hubs without receptor- or ligand-proximal evidence; ND, not demonstrated. Molecular docking alone was not considered sufficient evidence of direct binding (DB). Changes in receptor abundance (expression) were distinguished from changes in receptor phosphorylation. Suppression of NF-κB, inhibition of MMPs, antioxidant activity, induction of apoptosis, or nonspecific reduction in cytokine production, in the absence of receptor- or ligand-proximal evidence, were not classified as RTK modulation.

## Data Availability

No new data were created or analyzed in this study. Data sharing is not applicable to this article.

## Abbreviations

The following abbreviations are used in this manuscript:

AKT	Protein kinase B
AXL	AXL receptor tyrosine kinase
CAF	cancer-associated fibroblast
COX-2	cyclooxygenase-2
CXCL12	C-X-C motif chemokine ligand 12
CXCR4	C-X-C chemokine receptor type 4
ECM	extracellular matrix
EGF	epidermal growth factor
EGCG	epigallocatechin-3-gallate
EGFR	epidermal growth factor receptor
EMT	epithelial–mesenchymal transition
ERK	extracellular signal-regulated kinase
FAK	focal adhesion kinase
FGF	fibroblast growth factor
FGFR	fibroblast growth factor receptor
GAS6	growth arrest-specific 6
HGF	hepatocyte growth factor
HIF-1α	hypoxia-inducible factor 1 alpha
IGF	insulin-like growth factor
IGF1R	insulin-like growth factor 1 receptor
IL	interleukin
IL-1β	interleukin-1 beta
IL-6	interleukin-6
IL-8	interleukin-8
JAK	Janus kinase
JNK	c-Jun N-terminal kinase
MAPK	mitogen-activated protein kinase
MCP-1	monocyte chemoattractant protein-1
MEK	MAPK/ERK kinase
MET/c-MET	mesenchymal–epithelial transition factor
MMP	matrix metalloproteinase
mTOR	mechanistic target of rapamycin
NF-κB	nuclear factor kappa B
PDGF	platelet-derived growth factor
PDGFR	platelet-derived growth factor receptor
PGE2	prostaglandin E2
PI3K	phosphoinositide 3-kinase
Pro-EGCG	prodrug of epigallocatechin-3-gallate
RAS	rat sarcoma viral oncogene homolog
Rg3	ginsenoside Rg3
ROS	reactive oxygen species
RTK	receptor tyrosine kinase
Smad	small mothers against decapentaplegic
SRC	SRC proto-oncogene, non-receptor tyrosine kinase
STAT3	signal transducer and activator of transcription 3
TGF-β	transforming growth factor beta
TIMP	tissue inhibitor of metalloproteinase
TNF-α	tumor necrosis factor alpha
VEGF	vascular endothelial growth factor
VEGFR	vascular endothelial growth factor receptor
